# Effect of Carbetocin on Postpartum Hemorrhage after Vaginal Delivery: A Meta-Analysis

**DOI:** 10.1155/2022/6420738

**Published:** 2022-06-20

**Authors:** Xiaojuan Huang, Wanxing Xue, Jin Zhou, Cuiyi Zhou, Feiyan Yang

**Affiliations:** ^1^Department of Pediatrics, Haikou Maternal and Child Health Hospital, Haikou, Hainan 570203, China; ^2^Department of Obstetrics and Gynecology, Hainan Western Central Hospital, Danzhou, Hainan 571700, China; ^3^Haikou Maternal and Child Health Hospital, Haikou, Hainan 570203, China; ^4^Department of Obstetrics and Gynecology, Haikou Maternal and Child Health Hospital, Haikou, Hainan 570203, China; ^5^Department of Obstetrics and Gynecology, Hainan Modern Women and Children's Hospital, Haikou, Hainan 571100, China

## Abstract

**Background:**

The efficacy of oxytocin and carbetocin in preventing postpartum hemorrhage (PPH) in women with vaginal delivery has been controversial. This study is aimed at conducting a meta-analysis that compares the efficacy of carbetocin and oxytocin in the prevention of PPH among women with vaginal delivery.

**Methods:**

Literature was retrieved from PubMed, Medline, Embase, CENTRAL, and CNKI databases. The randomized controlled trials (RCTs) that compare the efficacy of carbetocin and oxytocin to prevent PPH were searched. Data from the included literatures were extracted by two researchers, including author, title, publication date, study type, study number, the incidence of PPH, number of patients requiring additional uterotonics, and number of patients requiring blood transfusion. Jadad scale was used to evaluate the quality of the included RCTs. The Chi-square test was adopted for the heterogeneity test. A fixed-effect model was used for analysis if heterogeneity did not exist between literatures. If heterogeneity exists between literatures, a random-effect model was used for analysis. The source of heterogeneity was explored by subgroup analysis and sensitivity analysis.

**Results:**

The incidence of PPH in the carbetocin group was lower than that in the oxytocin group (OR = 0.62, 95% CI (0.46, 0.84), *Z* = 3.14, *P* = 0.002). There was no heterogeneity among studies (*χ*^2^ = 7.29, *P* = 0.12, *I*^2^ = 45%) and no significant publication bias (*P* > 0.05). The proportion of women requiring additional uterotonics in the carbetocin group was lower than that in the oxytocin group (OR = 0.41, 95% CI (0.29, 0.56), *Z* = 5.34, *P* < 0.00001). There was no heterogeneity among studies (*χ*^2^ = 0.82, *P* = 0.84, *I*^2^ = 0%) and no significant publication bias (*P* > 0.05). There was no significant difference in the proportion of women needing blood transfusion between the carbetocin group and the oxytocin group (OR = 0.92, 95% CI (0.66, 1.29), *Z* = 0.46, *P* = 0.64). There was no heterogeneity among studies (*χ*^2^ = 3.06, *P* = 0.55, *I*^2^ = 0%) and no significant publication bias (*P* > 0.05).

**Conclusion:**

Carbetocin is superior to oxytocin in preventing PPH among women with vaginal delivery and can be widely used in clinical practice.

## 1. Introduction

Postpartum hemorrhage (PPH), as one of the leading causes of maternal death worldwide [1, 2], accounts for 27.1% of all maternal deaths [3]. The proportion of deaths caused by PPH is positively related to the income levels across countries [3–6]. The main cause of PPH is uterine atony [7]. Any factor affecting the normal contraction and retraction function of postpartum uterine muscle fibers can increase the amount of postpartum hemorrhage [8]. Pregnant women with multiple pregnancies, giant fetuses, polyhydramnios, placenta previa, and other conditions are prone to uterine asthenia PPH [9].

The induction of uterine contractions using clinical treatment could reduce the risk of PPH [10]. For example, oxytocin, as a uterine contractile agent, is widely used to prevent PPH [11]. The disadvantages of oxytocin including poor thermal stability and low-temperature transportation need cause the noneffective use in high temperature and humid environment [12]. Other conditions such as short half-life of oxytocin also lead to a frequent administration for patients. Overcoming the defects of oxytocin, another medical agent, carbetocin, showed better thermal stability and longer half-life [13, 14].

However, the efficacy of oxytocin and carbetocin in preventing postpartum hemorrhage in vaginal delivery has been controversial. Some studies [15] reported that application of carbetocin resulted in less PPH incident and lower amount of postpartum hemorrhage compared with oxytocin. In addition, the change of maternal systolic blood pressure after carbetocin administration was small. Other studies, however, noted that oxytocin and carbetocin showed similar therapeutic effects on PPH prevention [16]. To understand the similarity and differences, we conducted a meta-analysis to systematically evaluate the effects of oxytocin and carbetocin in PPH prevention in vaginal delivery.

## 2. Materials and Methods

### 2.1. Literature Extraction

Literature search was conducted in PubMed, Medline, Embase, CENTRAL, and CNKI databases. The searching criteria included (carbetocin) AND (postpartum haemorrhage OR PPH) AND (vaginal delivery OR vaginal birth). There were no restrictions on document language and publication time.

### 2.2. Literature Screening

Literature inclusion criteria: (1) the subjects were pregnant women with vaginal delivery; (2) the study included randomized control and experimental group; (3) the experimental group was given carbetocin, and the control group was given oxytocin; (4) the observed outcomes including at least one of the following: the incidence of PPH, the proportion of patients requiring additional uterotonics, and the proportion of patients receiving blood transfusion; (5) the type of study was randomized controlled study.

Literature exclusion criteria: (1) repeated reports and case reports; (2) the subjects included patients with cesarean section or undefined delivery method; (3) there was no control group in the study; (4) the balance of baseline data between the study group and the control group was poor; (5) the required data cannot be obtained, and the author of the literature cannot be contacted to supplement.

### 2.3. Data Extraction

In this paper, Huang and Xue jointly extracted the data information in the literature included in the analysis, including the author, title, publication time, research type, number of researchers, the incidence of PPH, number of patients requiring additional intrauterine tension, and the number of patients requiring blood transfusion. Data were unable to obtain in the literature can be obtained by contacting the author. When there were different opinions on literature data extraction, the two researchers discussed and reached an agreement.

### 2.4. Literature Quality Evaluation

The quality of included RCT studies was evaluated by Huang and Xue using the Jadad scale including the method of generating the random sequence, concealment of randomization, blinding, and the withdrawal rules.

### 2.5. Heterogeneity Test

The Chi-square test was used for the heterogeneity test. When *I*^2^ corrected by degrees of freedom was more than 50% or *P* < 0.1, it was considered that there was heterogeneity among published literatures, and a random effect model was used. Subgroup analysis was used to explore the causes of heterogeneity and sensitivity. If the source of heterogeneity could not be identified, the literature results were discussed without merging. When the *I*^2^ corrected by degrees of freedom was ≤50% and *P* ≥ 0.1, it was considered that there was no heterogeneity among the published literatures, and the fixed effect model was used.

### 2.6. Publication Bias Assessment

Egger test was used to evaluate the publication bias. *P* > 0.05 suggested no significant publication bias, and *P* < 0.05 indicated that there was a certain publication bias.

### 2.7. Statistical Method

In this study, Cochrane software RevMan5.3 was used for statistical analysis of the data. Statistical descriptions of effect sizes were performed using odds ratio (OR) values and a 95% confidence interval (CI). Two-sided *P* < 0.05 indicated statistical significance.

## 3. Results

### 3.1. Characteristics of Included Literature

**Figure 1:**
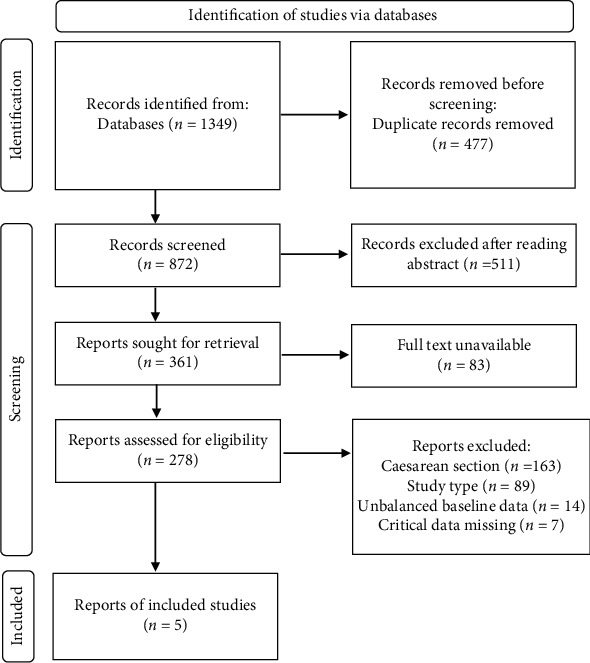
Flowchart of literature screening.

A total of 1349 literatures were retrieved according to method. Basing on screening criteria, 1344 literatures were excluded, and a total of 5 literatures were included in the study [15–19]. The flowchart of literature screening is shown in Figure 1. All 5 literatures were randomized controlled studies in English. This study included a total of 4631 pregnant women, among which, 2323 pregnant women used carbetocin and 2308 used oxytocin. The basic information of literature was summarized in Table 1, and Jadad score was listed in Table 2.

### 3.2. Comparison of PPH Incidence

**Figure 2:**
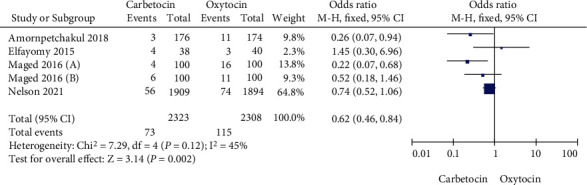
Forest chart compares the incidence of postpartum hemorrhage between the carbetocin and the oxytocin groups.

Five studies were included to compare the incidence of maternal PPH in the carbetocin group and the oxytocin group in our meta-analysis. Heterogeneity test showed that there was no heterogeneity among the five studies (Chi^2^ = 7.29, *P* = 0.12, *I*^2^ = 45%). Thus, the fixed-effect model was used for data consolidation. As shown in Figure 2, the incidence of PPH in carbetocin group was lower compared with that in oxytocin group (OR = 0.62, 95% CI (0.46, 0.84), *Z* = 3.14, *P* = 0.002). In addition, Egger test showed no significant publication bias among the studies (*P* > 0.05).

### 3.3. Comparison of the Proportion of Pregnant Women Using Extra Intrauterine Tension

**Figure 3:**
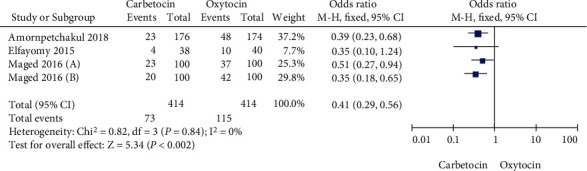
Comparison of the proportion of women in the carbetocin and oxytocin groups requiring additional uterine contractions.

In this meta-analysis, four studies compared the proportion of women in the carbetocin group and oxytocin group who needed additional intrauterine tension. Heterogeneity test showed that there was no heterogeneity among the four studies (Chi^2^ = 0.82, *P* = 0.84, *I*^2^ = 0%). Following, the fixed-effect model was used for data consolidation. The results showed, in Figure 3, that the proportion of pregnant women who needed additional intrauterine tension in the carbetocin group was lower than that in the oxytocin group (OR = 0.41, 95% CI (0.29, 0.56), *Z* = 5.34, *P* < 0.00001). Egger test showed no significant publication bias among the studies (*P* > 0.05).

### 3.4. Comparison of the Proportion of Parturient Receiving Blood Transfusion

**Figure 4:**
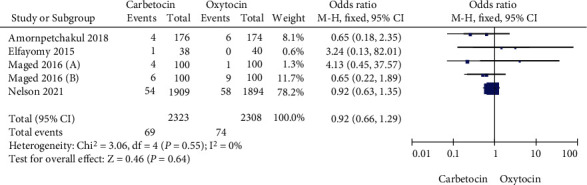
Forest map compares the proportion of pregnant women requiring blood transfusion in the carbetocin and oxytocin groups.

Five studies selected for this meta-analysis compared the proportion of pregnant women requiring blood transfusion in the carbetocin group and oxytocin group. Heterogeneity test showed that there was no heterogeneity among the five studies (Chi^2^ = 3.06, *P* = 0.55, *I*^2^ = 0%). The fixed-effect model was used for consolidation. There was no significant difference in the proportion of pregnant women requiring blood transfusion between carbetocin group and oxytocin group (OR = 0.92, 95% CI (0.66, 1.29), *Z* = 0.46, *P* = 0.64) as shown in Figure 4. Egger test showed no significant publication bias among the studies (*P* > 0.05).

## 4. Discussion

Our meta-analysis showed that PPH incidence and the use of additional intrauterine tensors in the carbetocin group were lower than those in the oxytocin group. There was no significant difference between the two groups in the proportion of parturient receiving a blood transfusion. This conclusion is consistent with some of the research results included in our analysis. Furthermore, Amornpetchakul et al. [17] studied singleton pregnant women with at least one PPH risk factor. Their results showed that the carbetocin group had a lower incidence of prevention of dystonic PPH and less use of additional uterine tension drugs than the oxytocin group. It was also noted that, although the blood loss after delivery was lower than that in the oxytocin group, the incidence of anemia was lower in carbetocin treated group. However, other studies such as Elfayomy [19] found no significant difference between the carbetocin group and the oxytocin group in blood loss, decreased hemoglobin level, and the proportion of pregnant women injected with additional uterine tension. Additionally, the reduction of the placenta was similar in both carbetocin and oxytocin groups. However, carbetocin advantaged in hemodynamic safety and stable blood pressure postadministration. Taken together, this study suggested to use carbetocin instead of oxytocin for placental delivery management. Maged et al. [15] conducted a prospective and double-blind study and found that PPH and the amount of bleeding in the carbetocin group were lower than those in the oxytocin group. Only slight decrease in maternal hemoglobin and hemodynamic changes was observed in carbetocin group. There was no significant difference between carbetocin and oxytocin in the incidence of nausea, vomiting, and dyspnea. Carbetocin was suggested to be more likely to cause maternal tachycardia; however, further research is needed. Maged et al. [15] studied high-risk pregnant women with PPH, and in another study, Maged et al. [18] presented that carbetocin had significant advantages in preventing PPH incidence compared with oxytocin. Still, there is no significant difference between maternal hemodynamic changes and side effects. Nelson et al. [16] considered that the effect of carbetocin was not inferior to oxytocin in the prevention of PPH, and the incidence of side effects was similar.

Tareef et al. [20] found no significant difference in using additional intrauterine tensors between the carbetocin group and the oxytocin group in pregnant women with vaginal delivery and elective cesarean section. The incidence of PPH in the carbetocin group was higher than that in the oxytocin group. The need of blood transfusion was more commonly seen in carbetocin treated group. The final conclusion of this study is inconsistent with our meta-analysis study. This study was excluded from meta-analysis study due to the objects included women undergoing elective cesarean section. Also, this study was a retrospective study. Another retrospective analysis [14] concluded that carbetocin and oxytocin had similar effects on the prevention of PPH, but carbetocin increased the incidence of placental retention.

In addition, some studies [21] indicated that carbetocin has an overwhelming advantage in economic benefits and costs for preventing PPH in pregnant women undergoing elective cesarean section. However, the same principle does not apply to women undergoing vaginal delivery. Studies such as Briones et al. [22] suggested that carbetocin is not a cost-effective choice for either vaginal delivery or cesarean section. The use of carbetocin needs to consider the economic cost, especially in low or middle-income families. For that purpose, oxytocin application is more widely used in low or middle-income countries.

Regarding to the limitations in this study, there are few randomized controlled trials comparing the effects of carbetocin and oxytocin on postpartum hemorrhage. High-quality randomized controlled trials are needed to validate the conclusions. Second, the age and underlying disease on drug efficacy were not taken into consideration due to the limited information. The clinical significance could be further deepened if multilayer analysis could be conducted.

In conclusion, carbetocin is superior to oxytocin in preventing PPH in vaginal delivery and can be popularized in the clinic.

## Figures and Tables

**Table 1:** Included literature characteristics.

Author and year	Study type	No. of patients		Outcomes	Drugs
		Carbetocin	Oxytocin		
Amornpetchakul et al. 2018 [17]	RCT	176	174	PPH, additional uterotonics, and blood transfusion	Oxytocin: 5 U; intravenous Carbetocin: 100 *μ*g; intravenous
Elfayomy 2015 [19]	RCT	38	40	PPH, additional uterotonics, and blood transfusion	Oxytocin: 50 IU; intravenous Carbetocin: 100 *μ*g; intravenous
Maged et al. 2016(A) [18]	RCT	100	100	PPH, additional uterotonics, and blood transfusion	Oxytocin: 5 IU Carbetocin: 100 *μ*g
Maged et al. 2016(B) [15]	RCT	100	100	PPH, additional uterotonics, and blood transfusion	Oxytocin: 5 IU; per os Carbetocin: 100 *μ*g; per os
Nelson et al. 2021 [16]	RCT	1909	1894	PPH, additional uterotonics, and blood transfusion	Oxytocin: 10 IU; intravenous Carbetocin: 100 *μ*g; intravenous

**Table 2:** Jadad score of included literatures.

Author and year	Randomization	Concealment of allocation	Double blinding	Withdrawals and dropouts
Amornpetchakul et al. 2018 [17]	1	1	2	1
Elfayomy 2015 [19]	2	1	2	1
Maged et al. 2016(A) [18]	2	2	2	1
Maged et al. 2016(B) [15]	1	2	1	1
Nelson et al. 2021 [16]	2	2	1	1

## Data Availability

The data used to support the findings of this study are included within the article.
